# Lifetime Paid Work and Mental Health Problems among Poor Urban 9-to-13-Year-Old Children in Brazil

**DOI:** 10.1155/2013/815218

**Published:** 2013-11-04

**Authors:** Isabel A. Bordin, Ivens H. Pires, Cristiane S. Paula

**Affiliations:** ^1^Departamento de Psiquiatria, Universidade Federal de São Paulo, Rua Borges Lagoa 570, 04038-030 São Paulo, SP, Brazil; ^2^Programa de Pós-Graduação em Distúrbios do Desenvolvimento, Universidade Presbiteriana Mackenzie, Rua da Consolação 930, Edifício 28, 01302-000 São Paulo, SP, Brazil

## Abstract

*Objective*. To verify if emotional/behavioral problems are associated with lifetime paid work in poor urban children, when taking into account other potential correlates. *Methods*. Cross-sectional study focused on 9-to-13-year-old children (*n* = 212). In a probabilistic sample of clusters of eligible households (women 15–49 years and son/daughter <18 years), one mother-child pair was randomly selected per household (*n* = 813; response rate = 82.4%). CBCL/6-18 identified child emotional/behavioral problems. Potential correlates include child gender and age, socioeconomic status/SES, maternal education, parental working status, and family social isolation, among others. Multivariate analysis examined the relationship between emotional/behavioral problems and lifetime paid work in the presence of significant correlates. *Findings*. All work activities were non-harmful (e.g., selling fruits, helping parents at their small business, and baby sitting). Children with lower SES and socially isolated were more involved in paid work than less disadvantaged peers. Children ever exposed to paid work were four times more likely to present anxiety/depression symptoms at a clinical level compared to non-exposed children. Multivariate modeling identified three independent correlates: child pure internalizing problems, social isolation, and low SES. *Conclusion*. There is an association between lifetime exposure to exclusively non-harmful paid work activities and pure internalizing problems even when considering SES variability and family social isolation.

## 1. Introduction

Asia-Pacific, Latin America, and the Caribbean regions continue to reduce child labor, while sub-Saharan Africa has witnessed an increase in both relative and absolute terms. Worldwide, child labor continues to decline, but only modestly, as 215 million children are still affected [1]. International labor standards define child labor by its consequences: it encompasses work that is mentally, physically, socially or morally dangerous and harmful to children and interferes with their schooling and personal development [2, 3]. Low school performance or school dropout due to child labor reduces job opportunities in adult life and limits the possibility of overcoming poverty. Also, physical injury and other health problems resulting from hazardous work conditions in childhood or adolescence tend to persist through adult life [4–6], while other negative effects of child labor involve impairment of physical, mental, and social development because of limited time for resting, playing, and studying, among other health and developmental problems [7].

In different countries, children exposed to poverty are frequently involved in paid or unpaid work activities for long periods of time with a negative impact on their education. More girls than boys are required to stay home to take care of younger siblings and to perform domestic chores, while more boys than girls are involved in paid work activities. Furthermore, work activities may be paid or unpaid, heavy or light, beginning at a younger or older age, with duration of a few hours or more than 20 hours per week, with a short or long duration in consecutive months, with a greater or lesser impact on children's education, and with greater or lesser risks for children's health [4, 5]. Therefore, it is important to clarify what types of work and what intensities of work are especially likely to impact health, education, or other aspects of child development.

The International Labour Organization, as a specialized agency of the United Nations, stipulates that national laws or regulations may permit the employment or work of persons aged 13/12 to 15/14 years on light work which is defined as not harmful to their health or development and not an obstacle to their attendance at school, participation in vocational orientation or training programs, or their capacity to benefit from the instruction received [8]. Therefore, child labor must be differentiated from light work, which is not necessarily detrimental to children's health or education when involving less than 14 working hours per week [8]. It is also important to consider that participation in nonhazardous activities (e.g., helping parents at home, assisting in family business, and earning pocket money outside school hours) provides children with skills and experience, preparing them to be productive members of society in adult life [2]. However, further research is needed to better examine the impact of light work on school attendance and on children's physical and mental health. 

This study is the first to examine—in a probabilistic household sample of 9-to-13-year-old children from a middle-income country (Brazil) living in an impoverish urban area—whether lifetime exposure to exclusively nonharmful paid work activities may be related to emotional and/or behavioral problems in children, when taking into account other potential correlates. Reasons for beginning to work for pay at an early age and differences in physical health and schooling between children exposed and nonexposed to exclusively nonharmful paid work during lifetime are also examined.

## 2. Methods

### 2.1. Study Design and Sampling

A cross-sectional study was conducted in southeast Brazil from April 2002 to February 2003, in a typical urban poor neighborhood of Embu, a city located in the great metropolitan area of São Paulo city, where more than 10% (19 million) of the Brazilian population live. In 2002, Embu had a population of 213,885 inhabitants, and 29.9% of them were less than 15 years of age [9].

The Brazilian Institute of Geography and Statistics randomly selected 24 clusters in the chosen neighborhood (where one of the local health centers was located), based on census units. In these clusters, all eligible households (those having a woman aged 15–49 years with at least one child <18 years of age) were identified. One mother-child pair was randomly selected per household. From the initial sample (*n* = 987), 813 mothers completed the study questionnaire (response rate: 82.4%). In this sample, children's age was 0–17 years, and children aged less than nine years never worked for pay. Because in Brazil 14-to-15-year-old persons are allowed to work as apprentices and it is legal to work if aged 16 years or more, the current study was restricted to children aged 9–13 years with complete information on all study variables of interest (*n* = 212). Only two subjects were excluded due to missing data.

### 2.2. Variables and Instruments

Any type of child lifetime paid work is the outcome of interest of this study. Potential correlates include variables from four domains: child (gender, age, emotional/behavioral problems, physical health problems, and school delay), mother (years of education, working status, and common mental health disorders), father (working status), and family (social isolation and socioeconomic status/SES). All variables were measured based on mother's report.

Trained interviewers applied the Child Behavior Checklist (CBCL/6–18) [10] to mothers to identify internalizing problems (anxiety/depression symptoms at a clinical level) and externalizing problems (breaking of rules and/or aggressive behavior at a clinical level) in children. The CBCL/6–18 is a standardized parent report screening questionnaire internationally used to identify emotional and behavioral problems in children and adolescents; it has adequate psychometric properties [10]. Validity of the Brazilian version of CBCL/6–18 (developed by Bordin et al. in 2002) is assumed based on studies that showed the high sensitivity of the Brazilian version of CBCL/4–18 compared to the “gold standard” ICD-10 psychiatric diagnosis [11, 12]. In this study, children with CBCL/6–18 *T* scores > 63 (above the 90th percentile according to American normative data) on internalizing or externalizing problem scales were classified as clinical cases, while borderline cases (*T* scores 60–63) were considered nonclinical.

Child physical health problems were defined by the presence of at least one of the following conditions: poor/bad general health, chronic health problem, permanent hearing/speech/vision problem, and/or physical deformity or handicap. Regarding child education, three variables were examined: (1) school delay (one or more years behind the grade expected for her/his chronological age), (2) grade retention (having repeated one or more school grades), and (3) school dropout (being currently out of school given that education is compulsory for all Brazilians aged 6 to 14 years). Data on maternal common mental health disorders were obtained applying the Self-Report Questionnaire (SRQ-20) [13] that was validated for the Brazilian population with a cut-off point >7 [14]. Social isolation was defined as the mother never counting on family members and counting very little/not at all on neighbors. Family SES was measured by a family economic classification questionnaire developed by the Brazilian Association of Research Companies to determine socioeconomic classes according to family purchase power [15]. The instrument is based on the number of home appliances, the existence of private bathrooms inside or outside the dwelling, the educational level of the head of the household, and the number of home employees working at least five days a week. In the current study, total scores were used to determine families' SES as a continuous variable. 

### 2.3. Ethical Procedures

The Research Ethics Committee of Universidade Federal de São Paulo approved this study. Trained interviewers obtained written informed consent and then administered the instruments to mothers, individually, at the local health center to guarantee privacy and safety of mothers and interviewers.

### 2.4. Statistical Analysis

Statistical analysis was performed using SPSS. Univariate analysis identified correlates individually associated with child lifetime paid work. Correlates with a *P* value <0.20 were selected to enter a logistic regression multivariate model to obtain adjusted odds ratio estimates. Multivariate analysis examined the relationship between emotional/behavioral problems and child lifetime paid work in the presence of other significant correlates. No high level of colinearity (phi > 0.5) was noted among the independent variables.

## 3. Results

The current study evaluated a population-based sample of 212 children (9–13 years) including 106 girls (50.0%) and 106 boys (50.0%). Lifetime paid work was reported by mothers of 17 children (8.0%). Rates among girls (6.6%) and boys (9.4%) did not differ statistically (*P* = 0.448). The majority of children involved in paid work activities during lifetime (58.8%) had a job related to commerce mostly in food markets or stores (Table 1).

The great majority (82.4%) of children involved in lifetime paid work was working for pay in the past 12 months. Almost half of children (47.1%) ever exposed to paid work had begun to work recently (past 12 months). When considering children that started to work more than one year ago, 75.0% of them were still working in the past 12 months. Children who worked for a longer period of time (9–36 consecutive months) (24.4%) worked for 4 to 38 hours per week. Children who worked 20–70 hours per week (58.8%) did that for less than a month to 12 consecutive months maximum. 

Reasons for beginning to work for pay at an early age included getting money for personal expenditures (41.2%), the pleasure obtained with the work activity (23.5%), helping the mother or father in their work (11.8%), the need of increasing family income (5.9%), and avoiding being alone (5.9%).

Because children with internalizing problems may have concomitant externalizing problems, the current study investigated the association of child lifetime paid work with three types of child mental health problems: pure internalizing problems, pure externalizing problems, and cooccurrence of both problems. Children ever exposed to paid work were four times more likely to present pure internalizing problems (OR = 3.9; 95% CI: 1.3–11.9; *P* = 0.011) compared to nonexposed children. Exposed and nonexposed children did not differ in pure externalizing problems and in the cooccurrence of internalizing and externalizing problems (Table 2). In addition, children with internalizing problems alone or in combination with externalizing problems had worked for a greater number of hours during lifetime than children without internalizing problems (Mann-Whitney *U* test: *P* = 0.046).

Child physical health problems, school delay, grade retention, and school dropout were not associated with child exposure to lifetime paid work (Table 2). Also, child gender, maternal education, mother working for pay, and father working for pay were not associated with child exposure to lifetime paid work (Table 3). Child age was not considered a correlate since similar mean scores were obtained by children exposed and nonexposed to lifetime paid work (11.2 ± 1.4 and 10.8 ± 1.4; *P* = 0.243). Regarding family SES, a lower mean score was obtained by children exposed to lifetime paid work compared to nonexposed peers (11.5 ± 3.6 versus 13.7 ± 4.3; *P* = 0.037) showing differences in family purchase power between the two groups. Furthermore, exposed children were almost six times more likely to belong to socially isolated families (OR = 5.8; 95% CI: 1.3–24.7; *P* = 0.036) compared to nonexposed children (Table 3). 

Multivariate analysis revealed that pure internalizing problems remained strongly associated with exposure to lifetime paid work (OR = 3.7; 95% CI: 1.2–11.8; *P* = 0.027) in the presence of social isolation and family SES (Table 4).

## 4. Discussion

According to Brazilian laws it is illegal to work before completing 16 years of age, except as an apprentice when aged 14 to 15 years [16]. In 2006, 4.5% of all Brazilian children aged five to 13 years were currently working in the last week of July [17]. However, because child work is usually seasonal and intermittent, rates based on work activities during lifetime would increase a great deal compared to rates observed in a shorter period of time. Therefore, not surprisingly the rate of lifetime paid work obtained by our study was 8.0%.

The current study found an association between lifetime exposure to exclusively nonharmful paid work activities and pure internalizing problems even when considering SES variability and family social isolation. Studies that investigate the association between child work and child mental health are extremely scarce in the literature. In Ethiopia [18], the Diagnostic Interview for Children and Adolescents was applied to two groups of children aged 5–15 years: 528 child laborers (domestic laborers attending school or assisted by an NGO, children involved in street vending, and children from small-scale private enterprises working in shops, garages, hotels, and handcrafts) and 472 noneconomically active school children. Cases and controls differed in the proportion of 11-to-15-year-old persons (70.8% versus 79.9%, *P* = 0.001) and in the proportion of males (51.3% versus 36.2%, *P* < 0.001). Compared to controls, child laborers were seven times more likely to present mood disorders (OR = 6.7, 95% CI: 2.2–22.5, *P* < 0.001) and three times more likely to present anxiety disorders (OR = 2.6, 95% CI: 1.3–5.5, *P* = 0.003). Cases and controls had the same probability of presenting disruptive disorders. The current study results are in accordance with these data from Ethiopia since children exposed to lifetime paid work had a greater probability of presenting pure internalizing problems (symptoms of anxiety/depression at a clinical level) compared to nonexposed children, while differences in the probability of presenting externalizing behaviors were not observed between the two groups. Regarding epidemiological studies involving Brazilian children, only one publication [19] was found evaluating the association between child work and child mental health problems. In south Brazil, a cross-sectional study conducted in low-income urban areas applied the CBCL/4–18 to identify emotional/behavioral problems in children involved in paid or unpaid work activities (production of market goods or services) or full-time domestic work in the past year. The authors found that 10-to-13-year-old persons exposed to domestic work (compared to nonworking children of same age) and 14-to-17-year-old persons that begun to work at an early age (compared to working peers of the same age who begun to work after 13 years) were more likely to be classified as clinical cases by the CBCL/4–18 total behavior problem scale [19]. Three other studies that investigated the association between labor-related variables and mental health problems could not be compared with the current study due to important methodological differences. Two of these studies [20, 21] examined samples including only working children with no comparison group, and two of them [21, 22] did not apply specific questionnaires commonly used in epidemiological studies to measure child mental health problems. The study conducted in Bangladesh [21] reported psychological problems in 40% of child laborers exposed to risky jobs (*n* = 80), mentioning that they became withdrawn, introvert, and uncommunicative without informing how psychological problems were measured. The study conducted in Lebanon [22] evaluated three groups of 10-to-17-year-old persons and noted that depression symptoms (based on neurobehavioral tests) were more frequent among children exposed to solvents in work in comparison to nonexposed working children and nonexposed schoolchildren. 

In the current study, the main reason for beginning to work for pay at an early age was getting money for personal expenditures. These results are in accordance with Brazilian national data, since 57.4% of 5-to-13-year-olds persons working for pay did not give their earned money to parents/caretakers, with this rate being even higher (68.2%) in the southeast region [17]. Also in the current study, other reason for beginning to work for pay early in life included the pleasure obtained with the work activity. This is also in accordance with Brazilian national data [17], since 63.2% of working 5-to-13-year-old persons were working because they wanted to, and not due to parents/caretakers' demands. Similar findings were obtained by a population-based study involving 4325 14-to-15-year-old persons from south Brazil, since around 80.0% of adolescents working for pay in the past 12 months reported that they worked by choice [23]. The authors also found that the proportion of working adolescents was higher among the poorer strata of the population (30.0%) compared to the more affluent (14.3%) [23]. The need of increasing family income was also mentioned by the current study informants as a reason for early child paid work, which is in agreement with the fact that children working for pay are more likely to belong to families of lower purchase power. Finally, avoiding being alone was also a reason for children starting to work early which is in accordance with the fact that children exposed to lifetime paid work were more likely to belong to socially isolated families compared to nonexposed children. Therefore, it is reasonable to hypothesize that in deprived communities working can be a way for the child to socialize and that the availability of other sources of social support may have a protective role for child labor, as observed in a Brazilian study that examined its relationship with social contextual factors [24]. When considering actions against child labor at the governments' level, social protection is increasingly seen as crucial in its prevention since poor families have less ability to absorb shocks from economic crises or sudden sickness or death in the family [25].

In the current study, school delay, grade retention, and school dropout were not associated with child exposure to lifetime paid work. This is in accordance with Brazilian national data on 5-to-13-year-old persons since the rate of school enrolment did not differ between working children (95.5%) and nonworking children (95.3%) [17]. However, different findings were obtained by a birth cohort study from south Brazil that evaluated 4452 children at age 11 years and noted that child paid work was associated with grade retention among boys but not among girls [26]. In addition, due to the reduced number of 5-to-13-year-old persons exposed to lifetime paid work in the current study sample, it was not possible to investigate the effect of the number of hours worked per week and the duration of work in consecutive months on schooling or mental health. However, it is reasonable to state that while limited light work is not necessarily detrimental to a child's formal education, long working hours, on the other hand, are likely to have more serious developmental consequences on the child. Long hours also mean less time available for children to exercise their rights to education and leisure [4]. Furthermore, long working hours may be detrimental to children's mental health. A study that evaluated 780 children (aged 9–18 years) from five Gaza Strip areas who were working in local workshops, farms, and small industries found that the total number of daily working hours was significantly associated with depression scores from the Depression Self-Rating Scale for Children [20].

According to the Regional Strategy for the Prevention and Elimination of Child Labour in Latin America for 2013–2020, broadening and deepening the knowledge base through research are still a key challenge to be addressed in the region in the coming years [25]. The current study has the strength of fulfilling a gap in the literature by examining the association of child exposure to exclusively nonharmful paid work activities during lifetime with child mental health, using a population-based sample of poor urban children from a middle-income country (Brazil) in an age range prohibited to work by national laws. However, study limitations must be recognized such as the reduced sample size that limited the power of statistical analysis to identify significant correlates and the study cross-sectional design that could not establish the time sequence in which internalizing problems and child work occurred. Longitudinal studies are needed to examine the long-term impact of exclusively nonharmful paid work activities early in life on the mental health and education of children and adolescents. 

## 5. Conclusions

There is an association between lifetime exposure to exclusively nonharmful paid work activities and pure internalizing problems even when considering SES variability and family social isolation. Even constituting a better option than the streets, working may put children under additional stress due to extra responsibilities taken early in life. Because social isolation can pressure children to start to work early, actions to increase opportunities for youth's involvement in sports, arts, music, and other cultural activities at the local community are highly relevant for prevention of child labor together with interventions to increase family income.

## Figures and Tables

**Table 1:** Type of paid work activities among boys and girls aged 9–13 years that ever worked for pay (*n* = 17) in Embu, São Paulo, Brazil.

Type of paid work	Girls (*n* = 7) *N* (%)	Boys (*n* = 10) *N* (%)	Total (*n* = 17) *N* (%)
*Commerce *			
Helper in mother's store	2 (28.5)	0 (0.0)	2 (11.7)
Helper in fruit and vegetable market	1 (14.3)	1 (10.0)	2 (11.7)
Fruit selling	0 (0.0)	2 (20.0)	2 (11.7)
Helper in a bakery store	0 (0.0)	1 (10.0)	1 (5.9)
Wrapping products in markets/stores	0 (0.0)	3 (30.0)	3 (17.7)
*Other *			
Baby sitter	1 (14.3)	0 (0.0)	1 (5.9)
Selling cans for recycling	1 (14.3)	0 (0.0)	1 (5.9)
Painting refrigerator magnets	1 (14.3)	0 (0.0)	1 (5.9)
Manual fare collection to transport people on a transit vehicle	1 (14.3)	0 (0.0)	1 (5.9)
Car painter helper in the father's repair shop	0 (0.0)	1 (10.0)	1 (5.9)
Flyer distribution	0 (0.0)	1 (10.0)	1 (5.9)
A variety of small jobs	0 (0.0)	1 (10.0)	1 (5.9)

**Table 2:** Health and education potential correlates of lifetime paid work among children aged 9–13 years (*n* = 212) in Embu, São Paulo, Brazil.

Potential correlates	Lifetime paid work		OR (95% CI)	*P *
	Yes	No		
	*N* (%)	*N* (%)		
Mental health problems				
None (reference)	7/140 (5.0)	133/140 (95.0)		
Pure internalizing problems^a^	7/41 (17.1)	34/41 (82.9)	3.91 (1.29–11.91)	0.011
Pure externalizing problems^a^	1/11 (9.1)	10/11 (90.9)	1.90 (0.21–17.01)	0.462
Both^a^	2/21 (9.5)	19/21 (90.5)	2.00 (0.39–10.35)	0.332
Physical health problems				
No (reference)	10/158 (6.3)	148/158 (93.7)		
Yes^b^	7/54 (13.0)	47/54 (87.0)	2.20 (0.80–6.11)	0.121
School delay				
No (reference)	14/186 (7.5)	172/186 (92.5)		
Yes^c^	3/26 (11.5)	23/26 (88.5)	1.60 (0.43–6.00)	0.445
Repeated one or more grades				
No (reference)	15/193 (7.8)	178/193 (92.2)		
Yes	2/19 (10.5)	17/19 (89.5)	1.40 (0.29–6.62)	0.654
School dropout				
No (reference)	16/208 (7.7)	192/208 (92.3)		
Yes	1/4 (25.0)	3/4 (75.0)	4.00 (0.39–40.70)	0.286

OR: odds ratio; CI: confidence interval.

^a^Compared to none.

^b^Presence of at least one of the following conditions: (1) poor/bad general health; (2) chronic health problem; (3) permanent hearing, speech, or vision problem; (4) physical deformity or handicap.

^c^One or more years behind the grade expected for her/his chronological age.

**Table 3:** Other potential correlates of lifetime paid work among children aged 9–13 years (*n* = 212) in Embu, São Paulo, Brazil.

Other potential correlates^a,b^	Lifetime paid work		OR (95% CI)	*P *
	Yes	No		
	*N* (%)	*N* (%)		
Child gender				
Female (reference)	7/106 (6.6)	99/106 (93.4)		
Male	10/106 (9.4)	96/106 (90.6)	1.47 (0.54–4.03)	0.448
Maternal education (years)				
8 or more (reference)	6/77 (7.8)	71/77 (92.2)		
0–7	11/135 (8.1)	124/135 (91.9)	1.05 (0.37–2.96)	0.927
Mother working for pay				
Yes (reference)	12/126 (9.5)	114/126 (90.5)		
No	5/86 (5.8)	81/86 (94.2)	1.71 (0.58–5.03)	0.329
Maternal mental health^c^				
No common disorders (reference)	12/149 (8.1)	137/149 (91.9)		
Common disorders present	5/63 (7.9)	58/63 (92.1)	0.98 (0.33–2.92)	0.977
Father working for pay				
Yes (reference)	12/143 (8.4)	131/143 (91.6)		
No^d^	5/69 (7.2)	64/69 (92.8)	1.17 (0.40–3.47)	0.774
Social isolation^e^				
No (reference)	14/202 (6.9)	188/202 (93.1)		
Yes	3/10 (30.0)	7/10 (70.0)	5.76 (1.34–24.72)	0.036

OR: odds ratio; CI: confidence interval.

^a^Child age (not included in this table) was not considered a correlate since similar mean scores were obtained by children exposed and non-exposed to lifetime paid work (11.2 ± 1.4 and 10.8 ± 1.4; *P* = 0.243).

^b^Family SES (not included in this table) was considered a significant correlate since a lower mean score was obtained by children exposed to lifetime paid work compared to non-exposed peers (11.5 ± 3.6 versus 13.7 ± 4.3; *P* = 0.037).

^c^According to the SRQ-20, common mental health disorders present (total score > 7) versus no common disorders (total score 0–7).

^d^One child who never worked for pay had no father.

^e^Defined as mother never counting on family members and counting very little/not at all on neighbors.

**Table 4:** Logistic regression model to estimate adjusted odds of correlates of lifetime paid work among children aged 9–13 years (*n* = 212) in Embu, São Paulo, Brazil.

Independent variables	OR (95% CI)	*P *
Child mental health problems^a^		
Pure internalizing problems^b^	3.70 (1.16–11.78)	0.027
Pure externalizing problems^b^	1.94 (0.20–18.91)	0.567
Both^b^	1.64 (0.30–8.85)	0.567
Social isolation^c^	7.07 (1.48–33.76)	0.014
Family socioeconomic status^d^	0.87 (0.76–0.99)	0.046

OR: odds ratio; CI: confidence interval.

^a^CBCL/6–18 scores in the clinical range.

^b^Compared to none.

^c^Mother never counting on family members and counting very little/not at all on neighbors: true (1) versus false (0).

^d^Continuous total score ranging from 2 to 25.
